# Effect of adjuvants on the humoral immune response to congopain in mice and cattle

**DOI:** 10.1186/1746-6148-8-63

**Published:** 2012-05-23

**Authors:** John Kateregga, George W Lubega, Erik B Lindblad, Edith Authié, Theresa Helen Taillefer Coetzer, Alain François Vincent Boulangé

**Affiliations:** 1 College of Veterinary Medicine, Animal Resources and Biosecurity, Makerere University, Box 7062, Kampala, Uganda; 2 Brenntag Biosector, Elsenbakken 23, DK-3600, Frederikssund, Denmark; 3 Risk Assessment Department, ANSES (French Agency for Food, Environmental and Occupational Health & Safety), 27/31 Avenue du général Leclerc, 94701, Maisons-Alfort Cedex, France; 4 School of Biochemistry, Genetics and Microbiology, University of KwaZulu-Natal, Private bag X01, Scottsville, 3209, South Africa; 5 CIRAD, UMR INTERTRYP, Centro de Biotecnologia-UEM, 01009, Maputo, Mozambique; 6 CIRAD, UMR INTERTRYP, F-34398, Montpellier, France

## Abstract

**Background:**

We investigated several adjuvants for their effects on the humoral immune response in both mice and cattle using the central domain of congopain (C2), the major cysteine protease of *Trypanosoma congolense*, as a model for developing a vaccine against animal trypanosomosis. The magnitude and sustainability of the immune response against C2 and the occurrence of a booster effect of infection, an indirect measure of the presence of memory cells, were determined by ELISA, while spectrofluorometry was used to determine and measure the presence of enzyme-inhibiting antibodies.

**Results:**

Mice immunized with recombinant C2 in TiterMax™, Adjuphos™, purified saponin Quil A™ or Gerbu™ showed the best response according to the evaluation criteria and the latter three were chosen for the cattle vaccination study. The cattle were challenged with *T. congolense* four and a half months after the last booster. Cattle immunized with recombinant C2 in purified saponin Quil A™ showed the best antibody response according to the measured parameters.

**Conclusions:**

We identified purified saponin Quil A™ as a good adjuvant for immunizations with C2. The results from this study will be useful in future attempts to develop an effective anti-disease vaccine against African trypanosomosis.

## Background

Trypanosomosis is a chronic wasting disease afflicting cattle and other livestock in sub-Saharan Africa and other parts of the world [1] and is caused by tsetse fly-transmitted protozoan parasites belonging to the genus *Trypanosoma*. Chemotherapy and chemoprophylaxis have not proved to be very effective against the disease due to two main reasons:- the prohibitively high cost of trypanocidal drugs to resource-poor farmers [2] and the development of drug resistance [3]. Efforts to develop a vaccine directed against the immunodominant variant surface glycoprotein (VSG) have not been successful due to antigenic variation [4]. Vaccination efforts have shifted towards the characterization of invariant antigens that are accessible to the specific antibodies such as glycerophosphate-inositol anchor [5], flagellar pocket (FP) fractions [6] and tubulin [7].

An alternative approach based on an ‘anti-disease’ rather than an anti-parasite vaccine strategy was proposed following observations that trypanotolerant African taurine cattle, which have a natural ability to control trypanosome infection [8], develop more prominent antibody responses to congopain *-* the major cysteine protease (CP) of *T. congolense* than trypanosusceptible breeds [9]. It was hypothesized that congopain may therefore play a role in pathology of the disease, and antibody-mediated inhibition of this enzyme may contribute to mechanisms of trypanotolerance [10]. It hence constitutes a promising candidate vaccine in the sense that immunization of trypanosusceptible cattle with appropriate forms of cysteine proteases may confer a degree of resistance to the disease [11,12].

The goal of vaccination is to stimulate a strong, protective and long lasting immune response to the administered immunogen. To achieve these objectives, potent adjuvants are required to make the antigen sufficiently immunogenic to initiate a potent immune response [13]. While adjuvants were initially thought of as agents capable of promoting and sustaining antibody response, new evidence has shown that adjuvants influence the titer, isotype and avidity of antibody, and affect the properties of cell-mediated immunity [14]. Adjuvants can therefore be used to improve the immune response to vaccine antigens for several different purposes, *i.e.* increasing the immunogenicity of weak antigens; enhancing the speed and duration of the immune response; decreasing the dose of antigen in the vaccine to reduce costs or decreasing the number of immunizations [15].

This study was carried out within the framework of the European Union-funded INCO-DEV Trypadvac2 project (PL003716), the overall objective of which is to limit pathology associated with trypanosomal infections through development of an anti-disease vaccine. We investigated the effects of an array of adjuvants on the immune response in mice, and selected the best three adjuvants for a similar investigation in cattle using recombinant congopain (C2) [16] as a model antigen. The general objective of the study was to find the best adjuvant(s) giving an adequate response to be used in anti-disease vaccine trials. The rationale behind using C2 as a model lies in the fact that it has been shown to be poorly immunogenic, is a highly conformational, compact protein and its protective epitopes are dimer-specific [16]. In our study, response to C2 was monitored using four key parameters, namely 1/magnitude of the humoral immune response to immunization, 2/sustainability of the response after the last booster, 3/presence of enzyme-inhibiting antibodies and 4/occurrence of a booster effect of infection, an indirect measure of the presence of memory cells, whereby the re-exposure of the animals’ immune system to the immunizing antigen released during infection triggers an activation of the specific immune response.

We report here the detailed comparison of seven adjuvants of various types in mice and three in cattle, following the above parameters, leading to the selection of saponin-based Quil A [17] for subsequent immunization/challenge experiments in the development of an anti-disease vaccine against African trypanosomosis.

## Methods

### Materials

Congopain central domain (C2) was prepared as described in [16]. The antigen, expressed in *Pichia pastoris* as a recombinant antigen, was purified from the culture medium using a combination of three phase partitioning (TPP) [18] and ion-exchange and molecular exclusion chromatography. The batch used for immunization was freshly purified, and the dimeric conformation assessed thoroughly before use [16].

The adjuvants used in the study were; GERBU Adjuvant™ type 100 #3100 [GERBU Biotechnik GmbH, Gaiberg, Germany]; Adjuphos™ (aluminum phosphate) batch 8987 [Brenntag Biosector, Frederikssund, Denmark]; Alhydrogel™ (aluminum hydroxide) batch 3666 [Brenntag Biosector]; TiterMax™ [Sigma-Aldrich Chemie GmBH, Munich, Germany]; Freund’s complete adjuvant (FCA) [Sigma-Aldrich Chemie GmBH]; purified saponin Quil A™ batch L-77-238 [Brenntag Biosector] and α_2_-macroglobulin (α_2_M). α_2_M was purified from bovine plasma and complexed with recombinant C2 at a 1:1 molar ratio as described in [19]. Freund’s incomplete adjuvant (FIA) [Sigma-Aldrich Chemie GmBH] was used during the booster immunizations for the FCA groups.

Swiss mice, aged 4–6 weeks at the start of the study, were housed in the Laboratory Animal experimental facilities of the School of Veterinary Medicine of Makerere University, Uganda, in cages containing 5 animals each. Mice were fed on normal rodent pellets and water provided ad-libitum.

Trypanosome-free female Ankole cattle (aged 8–18 months) were used in the cattle study. Sera from a number of candidate animals for the experiment were screened for any prior exposure to trypanosomal antigens using standard ELISA [20]. In addition, ELISA was performed with cattle sera on C2-coated microtiter plates (Maxisorp, Nalge NUNC Int, Rochester, IL, USA) to check for non-specific reactivity to C2. Sera from trypanosome-infected cattle served as a positive control. Only anti-C2 negative animals were selected for the study. The cattle, initially acclimatized for 8 weeks, were kept indoors for the duration of the experiment in the fly-proof Large Animal Experimental Facilities of the College of Veterinary Medicine, Animal. Resources and Biosecurity of Makerere University, Uganda. They were fed on hay and concentrate, with mineral and vitamin supplements occasionally provided. Water was made available ad-libitum. For both mouse and cattle experiments, animal ethics clearance was obtained from the Higher Degrees Committee of the College of Veterinary Medicine, Animal Resources and Biosecurity of Makerere University, Uganda.

The trypanosome stabilates used in the experiments were obtained from the International Livestock Research Institute (ILRI), Nairobi, Kenya. *Trypanosoma congolense* IL3000 strain [21] was used in cattle and *T. congolense* IL1180 parasites [22] in mice since IL3000 was deemed too pathogenic for rodents [23].

### Mice immunization, trypanosome challenge and blood-sample collection

A total of 90 mice were randomly assigned to nine groups of 10 mice each, 5 males and 5 females, kept separately. Seven groups were immunized with C2 in combination with adjuvant, one group with C2 alone and a last group with PBS with neither adjuvant nor antigen (negative control). The mice received an initial immunization of 20 μg of C2 followed by two boosters of 10 μg of C2 each, at monthly intervals. For GERBU™, TiterMax™ and FCA the antigen and adjuvant were prepared in a ratio of 1:1 (v/v), 100 μl per mouse, thoroughly mixed/emulsified and administered sub-cutaneously (SC) in two sites. For aluminum-based adjuvants, 50 μl of antigen was mixed with 50 μl of adjuvant suspension, corresponding to approximately 250 μg of metallic aluminum per dose for Adjuphos™ and 500 μg for Alhydrogel™. For the mice immunized with Quil A™, 20 μg of C2 was mixed with 20 μg of adjuvant using a 2 mg/ml stock in a 1:1 v/v ratio giving a final concentration of adjuvant of 1 mg/ml, as recommended by the manufacturers, in a total volume of 100 μl per dose. The α_2_M-C2 complexes corresponding to 20 μg of C2 were diluted to 100 μl per mouse in PBS and injected as described previously [19]. Adjuvants were used at all booster immunizations but FIA was used for the boosters for the FCA group.

The mice were bled weekly from the tail (1–1.5 ml of blood per group) for the first 3 months and later once a month for the next 6 months to determine the magnitude and sustainability of the antibody response respectively. Blood samples from individual mice were pooled according to the different experimental groups to ensure sufficient serum volumes for analyses and to determine the average response of all the mice in each group, rather than individual animals’ responses. The samples were centrifuged and sera were collected and stored at −20°C until further analysis. Large amounts of sera (1.5-2 ml per group) were also collected at the time of the hypothetical peak of the response *i.e.* 2 weeks post last booster and used for immunoglobulin purification for enzyme inhibition assay purposes. Six months after the last booster, mice were challenged with *T. congolense* (10^3^ parasites per mouse administered intra-peritoneally). For the infection, infected mouse blood diluted in PBS was used. The infected blood was obtained from Swiss mice which had earlier been infected with 10^3^ *T. congolense* IL1180 parasites per mouse and their parasitaemia monitored daily. The trypanosome challenge experiment was aimed at determining the presence of a ‘booster effect’ of infection on the immune response (memory) in the immunized mice when compared with the control mice that were not immunized but infected with parasites.

### Cattle immunization, challenge and sample collection

Twenty cattle were randomly assigned to five groups of 4 animals each. Three groups were immunized with C2 in combination with one of 3 different adjuvants (GERBU™, Adjuphos™, Quil A™). Positive and negative control groups were immunized with C2 in FCA and C2 in PBS without adjuvant respectively. FCA was used as positive control since its ability to enhance the immune response in cattle was previously proven (Boulangé, *unpublished observation*) and for this group, FIA was used during the boosters. Cattle were initially immunized with 100 μg of C2 per animal, followed by two booster immunizations (50 μg per animal), done at two-week intervals after the initial immunization. The antigen/adjuvant mixtures were prepared as a 1:1 (v/v) ratio in a total volume of 3 ml per dose, directly for FCA, GERBU™ and Adjuphos™ (for the latter giving a 7.35 mg metallic aluminum per dose), and using a 2 mg/ml solution for Quil A™, *i.e.* 1 mg/ml, or 3 mg per dose. Animals were injected SC in the dew-lap, at two sites.

Blood from the individual animals (10 ml) was collected from the jugular vein at weekly intervals for the first 3 months of the experiment using serum separator tubes containing clot activator and serum separator gel (Yanvac™, Xiamen, Fujian, China). Sera were separated for ELISA purposes. A larger bleed (25 ml per cattle) was done 15 days post last booster, the alleged peak of response, for purification of IgGs for enzyme inhibition assay purposes. Thereafter blood collection was done once a month for 4½ months to monitor the sustainability of the antibody response. The sera were stored at −20°C until further analysis.

Four and a half months after the last booster, animals were infected with *T. congolense* IL3000 (10^3^ parasites per animal), intra-dermally using an intra-dermal syringe. Trypanosomes originated from fresh, PBS-diluted infected mouse blood. After challenge, the animals were monitored daily to determine the pre-patent period. Thereafter blood samples were also collected from the jugular vein twice a week in EDTA tubes to determine PCV (using the hematocrit method) and parasitaemia using wet smears or the hematocrit centrifugation-buffy coat technique [24]. Sera were collected once a week from one of the two blood samples to monitor the dynamics of the immune response and determine presence of a ‘booster effect of infection’. The degree of effectiveness of the adaptive immune response in providing protective immunity to infection was also assessed through monitoring PCV values twice a week. All the animals were treated with diminazene aceturate (Veriben®, CEVA Santé Animale, Libourne, France) 8 weeks post-infection. Post-treatment serum samples were collected 2 weeks later.

### Determination of anti-C2 antibody response by ELISA

The magnitude and sustainability of the immune response was determined by ELISA. Wells of ELISA plates (Corning-Costar, Lowell, MA, USA) were coated with 100 ng of C2 in 100 μl of 50 mM carbonate buffer, pH 9.6 and incubated overnight at 4°C. After discarding the contents of the wells, non specific binding sites were blocked with 100 μl of 0.5% (w/v) BSA (Fraction V, Sigma-Aldrich) in PBS by incubation at 37°C for 1 h. Sera, 1:100 dilution in 0.5% (w/v) BSA in PBS, were added and incubated for 2 h at 37°C. The wells were rinsed with PBS (pH 7.4) and washed twice with PBS containing 0.05% (v/v) Tween-20. For mouse sera, 100 μl of rabbit anti-mouse horse radish peroxidase (HRPO)-conjugated IgG (Sigma-Aldrich #A9044), diluted 1:5,000 in 0.5% (w/v) BSA in PBS, was used. For cattle sera, 100 μl of rabbit anti-bovine HRPO-conjugated IgG (Sigma-Aldrich #A5295) at a 1:20,000 dilution in 0.5% (w/v) BSA in PBS was added to each well and incubated for 1 h at 37°C. Substrate, 100 μl of 5 mM ortho-phenylenediamine (OPD) in 50 mM citrate-phosphate buffer (pH 5.0) containing 0.04% H_2_O_2_, was added after the washing step. Plates were incubated for 10–15 min at 25°C. The reaction was stopped with 100 μl of 3 N H_2_SO_4_ and optical densities (ODs) were determined at 492 nm using an ELISA reader (Finstruments™ Microplate Reader, MTX Lab Systems, Inc, Vienna, VA, USA).

### IgG purification

IgG was purified from mouse sera by using Protein A-Sepharose (SigmaAldrich). The pH of the serum was adjusted to 8.0 by mixing with ^1^/_10th_ volume 1 M Tris–HCl buffer, pH 8 before loading on the Protein A-Sepharose. After washing the column with 10 column volumes of 100 mM Tris–HCl buffer (pH 8.0), bound antibody was eluted with 100 mM glycine-HCl buffer, pH 3.0. The eluate was collected in 1 ml fractions containing 100 μl of 1 M Tris–HCl buffer, pH 8.5 and mixed gently to neutralize the pH. The IgG containing fractions were identified by measuring the absorbance at 280 nm.

IgG was purified from cattle sera by precipitation using polyethylene glycol (PEG) M_r_ 6000 [25]. Sera from the different animals in each group (according to adjuvant), taken 2 weeks post-last booster were pooled into 15 ml tubes. One volume of cattle serum was diluted with two volumes of borate buffered saline, pH 8.6 (composition - 2.16 g boric acid, 2.19 g NaCl, 0.7 g NaOH and 0.62 ml of 37% (v/v) HCl dissolved in 950 ml of distilled H_2_O). Solid PEG (M_r_ 6000) was added to the diluted serum to 14% (w/v) and dissolved with constant gentle stirring. The mixture was centrifuged (12000 × g; 10 min; 25°C) and the pellet redissolved in the original serum volume, using 100 mM Na_2_PO_4_ buffer, pH 7.6, containing 0.02% (w/v) NaN_3_. PEG was once again added to 14% (w/v), dissolved with stirring and centrifuged (12000 × g; 10 min; 25°C). The resulting pellet was re-dissolved in half the original serum volume using 100 mM Na_2_PO_4_ buffer, pH 7.6 containing 60% (v/v) glycerol and stored at −20°C until further analysis. The IgG concentrations were estimated using an extinction coefficient of $\sum_{1\text{mg/ml}}^{280\text{nm}}=1\text{.}43$[26].

### Inhibition of C2 activity

The *in-vitro* inhibition of C2 activity by purified IgGs from mice and cattle was assessed using assays for the hydrolysis of a fluorogenic peptide substrate. Ten ng of active C2 (determined by active site titration with E-64) [27] diluted in 75 μl of 0.1% (v/v) Brij-35 was mixed with 75 μl of the different IgG solutions diluted in C2 assay buffer (100 mM Bis-Tris buffer, pH 6.4, 4 mM Na_2_EDTA and 0.02% (w/v) NaN_3_) without dithiothreitol (DTT). The inhibition assay was performed at three different dilutions of IgG (*i.e.* 250 μg/ml, 125 μg/ml and 62.5 μg/ml for mouse IgGs [final amount of 6.25, 3.125, and 1.56 μg per well respectively] and 1000 μg/ml, 500 μg/ml and 250 μg/ml for cattle IgGs [final amount of 25, 12.5, and 6.25 μg per well respectively]) collected 2 weeks after the last booster immunisation. The mixtures (in microfuge tubes) were incubated overnight at 4°C. A 50 μl aliquot from the C2-IgG mixture was added per well of Fluoronunc Maxisorp 96-well plates (eBioScience, San Diego, CA, USA), followed by addition of 25 μl of assay buffer containing 8 mM DTT and activated for 1 min at 37°C. The dipeptide fluorogenic substrate Z-Phenylalanyl-Arginine-7-Amino-4-methyl coumarin (25 μl of 20 μM per well) was added immediately. Fluorescence was measured using a spectrofluorometer (Fluostar Optima, BMG Labtech GmbH, Offenburg, Germany) using excitation at 360 nm and emission at 460 nm. The slopes of the linear activity curves for the residual C2 were determined (Table 1). 

**Table 1:** Computation of percent inhibition of C2 activity

**IgG conc. [μg/ml]**	**Slope of linear plot of residual C2 activity**	**Slope of linear plot of C2 activity assayed with non- immune IgGs**	**Percent inhibition of C2 activity**
250	4.6078	8.0837	43
125	4.6724	7.1883	35
62.5	5.2348	7.3729	29

The slopes of the linear plots for the residual C2 activity were determined in a spectrofluorometer using excitation at 360 nm and emission at 460 nm. Percent inhibition of C2 activity was determined using the formula:$\text{Percent inhibition}\;\text{of C}2=\frac{\text{slope}\;\left(\text{C}2+\text{non}-\text{immune IgG}\right)-\text{slope}\;\left(\text{C}2+\text{immune IgG}\right)\times 100}{\left(\text{C}2+\text{non}-\text{immune IgG}\right)}$

Immune IgGs were obtained from mice or cattle immunized with C2 with adjuvant while non-immune IgGs were obtained from mice or cattle before immunization. The table shows the computation of percent inhibition of C2 activity by IgGs from mice immunized with C2 in Adjuphos™ adjuvant as an example.

### Statistical analysis

The Student’s *t* test was used to determine statistically significant differences in ODs or PCV between any two adjuvant groups of animals.

## Results

### Anti-C2 response in mice

To assess the anti-C2 response in mice, OD was determined instead of titer as titer determination would require large volumes of sera and running a large number of assays. The objective of the mice experiment was to provide a simple evaluation of the anti-C2 response in different adjuvant groups in order to choose the best adjuvants for the cattle study. The magnitude and sustainability of the response, presence of enzyme inhibiting antibodies post-immunization and occurrence of a booster effect of infection following challenge were evaluated.

**Figure 1:**
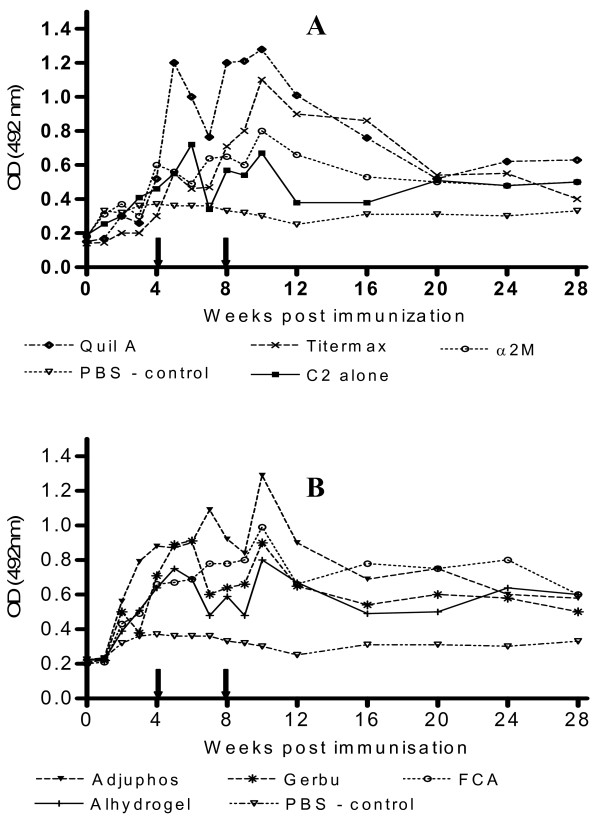
**ELISA of anti-C2 antibody response of mice immunized with C2 in different adjuvants.** Mice were initially immunized SC with 20 μg of C2 in Adjuphos™ GERBU™, Alhydrogel™, TiterMax™, FCA, Quil A™ or α_2_M adjuvants. The first and second booster immunizations (10 μg each) were done at 4 and 8 weeks post initial immunization respectively, indicated by arrows on x-axis. ELISA plates were coated with overnight with C2 at 4°C. OPD was used as substrate and absorbances were read at 492 nm. Each point is the average of duplicate wells.

Generally, peak antibody responses were observed around weeks 6 and 10 post initial immunization *i.e.* 2 weeks after the first and second booster immunizations respectively (Figure 1). The duration of the antibody response was evaluated by comparing the magnitude of response after week 10. Mice immunized with C2 combined with either Quil™, Adjuphos™, TiterMax™, FCA and GERBU™ adjuvants showed the best results in terms of magnitude and duration of response.

**Figure 2:**
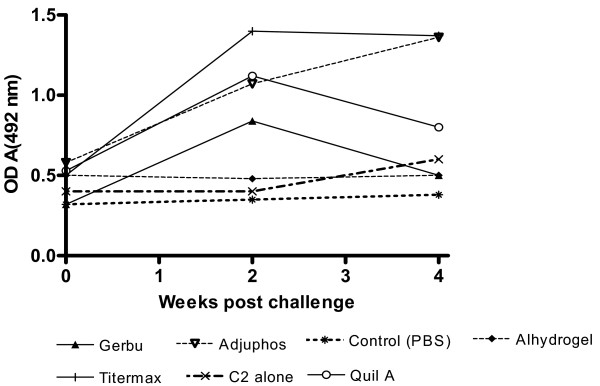
**ELISA of anti-C2 response of mice challenged with*T. congolense. ***Six months after the last booster, all the mice were challenged with *T. congolense* IL1180 (10^3^ parasites per mouse) administered intra-peritoneally. Day 0 was the day of infection. Post challenge antibody response was evaluated ELISA using sera collected just before challenge (week 0), and at weeks 2 and 4 post-challenge.

Figure 2 shows that there was a booster effect on the antibody response following infection of mice immunized with C2 in TiterMax™, Quil A™, Adjuphos™ and GERBU™ adjuvants. The booster effect was however, rather short lived for Quil A™ and GERBU™. The presence of a booster effect of infection could not be determined for the groups immunized with α_2_M or FCA because a number of animals in these groups had died during the course of the experiment, such that the remaining numbers did not constitute a statistically representative sample.

**Figure 3:**
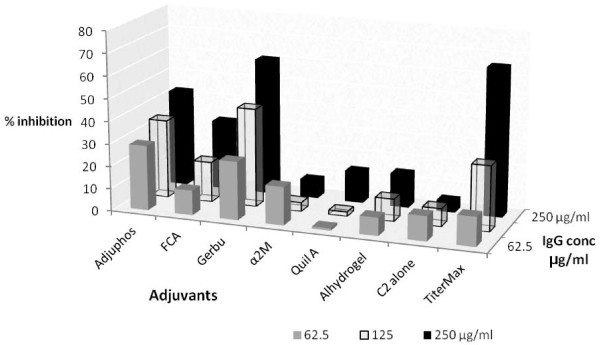
**Percent inhibition of C2 activity by IgGs purified from sera of immunized mice.** Inhibition assays were used to assess the ability of immune IgGs to inhibit C2 activity *in-vitro*. IgGs were purified by affinity chromatography on Protein A-Sepharose, from serum collected 2 weeks post-last booster. The assays were performed at three different concentrations of IgG *i.e.* 250, 125 and 62.5 μg/ml (final amount of 6.25, 3.125, and 1.56 μg per well respectively) and with 3.3 ng C2 per well. The C2-IgG mixtures were incubated overnight at 4°C. Residual C2 activity was measured after activation with 8 mM DTT. The fluorogenic substrate used was 20 μM Z-Phe-Arg-AMC and the fluorescence recorded (excitation 360 nm and emission 460 nm). The percent inhibition of C2 activity was determined from the slopes of the linear plots of residual C2 activity.

Purified IgGs from sera of TiterMax™, GERBU™ or Adjuphos™ mice groups showed the highest levels of inhibition of C2 activity *in vitro* (Figure 3). The highest inhibition (66%) was obtained with IgGs purified from sera of mice immunized with C2 with TiterMax™ adjuvant at an IgG concentration of 250 μg/ml; followed by GERBU™ (61%) and Adjuphos (43%). However, IgGs from the GERBU™ group showed the greatest degree of inhibition when the IgG concentration was halved to 125 μg/ml (*i.e.* 44% inhibition). At a lower IgG concentration *i.e.* 62.5 μg/ml, Adjuphos appears to be marginally the best (Figure 3).

In summary, TiterMax™, Adjuphos™, GERBU™ and Quil A™ were deemed the ‘best’ adjuvants in the mice experiment and the latter three were selected for the cattle study (see below). The GERBU™ and Adjuphos™ groups showed good peak antibody responses, a booster effect of infection on antibody levels and a high level of inhibition of C2 activity *in vitro.* The Quil A™ group showed a high peak response and a booster effect of infection. Despite the good results showed by the TiterMax group (good response, booster effect of infection and C2 inhibition), this adjuvant was not chosen for the cattle study since it was very expensive and not ideal for use in a cost-friendly anti-disease vaccine.

### Anti-C2 response in cattle

**Figure 4:**
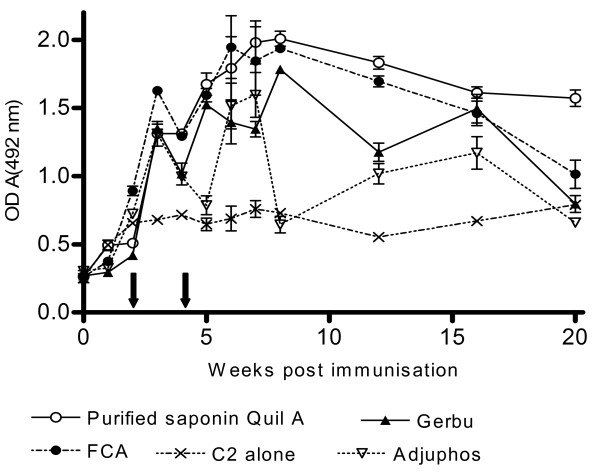
**ELISA of anti-C2 response in cattle after immunization.** Cattle were each immunized SC with 100 μg of C2 in GERBU™, Adjuphos™, Quil A™ or FCA. A control group was immunized with C2 alone. Two booster immunizations (50 μg per animal) were done at 2 weekly intervals after the initial immunization as indicated by the arrows on the x-axis. Reactivity towards C2 was monitored by ELISA.

Like in the mouse experiment, the main parameters evaluated were magnitude and sustainability of the antibody response, presence of C2 inhibiting antibodies *in-vitro* and a booster effect of infection on antibody levels. Two peaks in the immune response were observed *i.e.* around 2 weeks post first booster and 2–4 weeks post second booster (Figure 4). Cattle immunized with C2 in Quil A™ showed the best antibody response. This was followed by cattle immunized with C2 in FCA, and C2 in GERBU Adjuvant™ (Figure 4). The differences between FCA and Quil A™ or GERBU™ and Quil A groups were not significant (p >; 0.05). However there was a significantly higher response (by OD) for the Quil A™ group when compared with the Adjuphos™ group (p <; 0.05). The antibody response was best sustained at high levels in animals immunized with C2 in Quil A™ (Figure 4).

**Figure 5:**
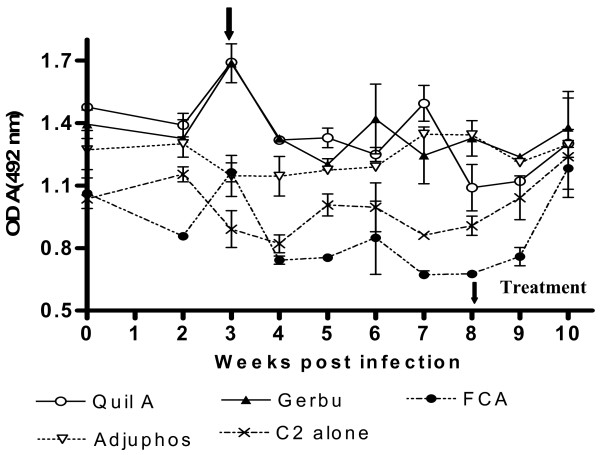
**ELISA of anti-C2 antibodies produced in cattle post infection.** Four and a half months after the last immunization booster, all the cattle were infected intra-dermally with *T. congolense* IL3000 (10^3^ parasites per animal). The pre-patent period was 9–11 days. The arrows show the ‘booster effect’ of infection around 2 weeks post patency. The arrow on the x-axis shows the time of treatment with diminazene aceturate.

The pre-patent period for the appearance of parasites in circulation after challenge was 9–11 days. Following infection, the antibody response (as measured by OD) remained highest for animals immunized with C2 in Quil A™, Adjuphos™ or GERBU™ adjuvants (Figure 5). However the ODs obtained post-challenge for animals immunized with C2 in FCA were not significantly different from those obtained for C2 alone (p >; 0.05) indicating that the immune response for this group had waned. A short-lived booster effect of infection was visible with GERBU™ and Quil A™ groups around week 3 post challenge (Figure 5), corresponding to 10–12 days after the appearance of parasites in circulating blood. There was also an increased response in all the cattle groups after diminazine treatment probably due to an antibody response to the release of antigens from the dead trypanosomes.

**Figure 6:**
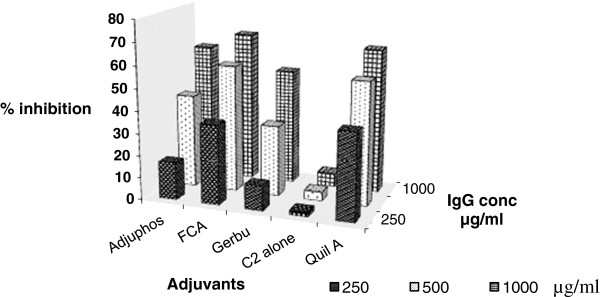
**Percent inhibition of C2 activity by cattle IgGs.** Inhibition assays to assess the ability of immune IgGs to inhibit C2 activity *in-vitro* were performed at IgG conc. of 1000, 500 and 250 μg/ml (25, 12.5, and 6.25 μg per well respectively). The amount of C2 used in the assay was 1.65 ng active enzyme per well. The C2-IgG mixtures were incubated overnight at 4°C and residual C2 activity was measured after activation with 8 mM DTT. The fluorogenic substrate used was 20 μM Z-Phe-Arg-AMC and the fluorescence recorded (excitation 360 nm and emission 460 nm). Percent inhibition of C2 activity was determined from slopes of the linear plots of residual C2 activity.

The *in-vitro* C2 inhibition assays showed that IgGs obtained from the Quil ATM, FCA, Gerbu™ and Adjuphos™ groups of cattle all showed high inhibition of the activity of C2 (Figure 6), in the range of 60% to 70% inhibition. However at lower concentration of IgGs, Quil A™ showed the highest inhibition of C2 activity, with up to 40% inhibition at an IgG concentration of 250 μg/ml (6.25 μg per well). Almost no inhibitory effect was observed on C2 activity for the group immunized with C2 alone. As shown in Figure 6, C2 inhibition increased with IgG concentration.

**Figure 7:**
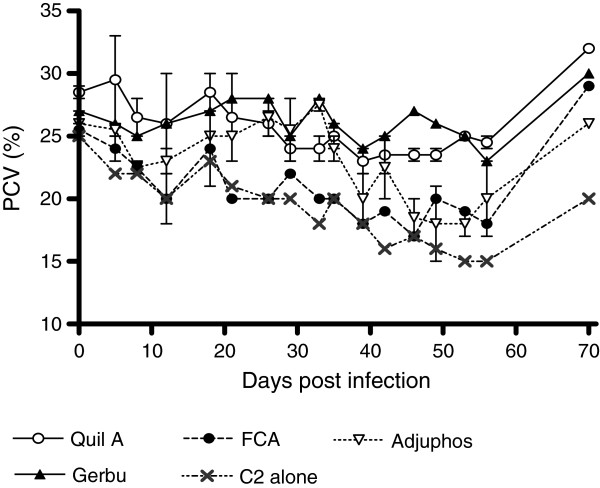
**Mean PCV of experimental cattle following challenge with*T. congolense. ***Following challenge with *T. congolense* IL3000 parasites, PCV was determined weekly using the hematocrit method, for 10 weeks. Animals were treated at week 8 (day 56), and a last sample collected 2 weeks later.

In addition to the four key antibody parameters, PCV was determined to evaluate the degree of protection offered by the immunization. As shown in Figure 7, PCV was highest for animals immunized with C2 in Quil A™ (which also showed the highest antibody response and enzyme inhibition) and least for animals immunized with C2 alone, which showed the lowest antibody response. This indicates that antibodies produced following immunization with C2 in adjuvant could be more effective in alleviation of pathogenic effects like anaemia. There were no parasitaemia differences between groups. However, we did not consider parasitaemia a key parameter for choice of best adjuvant given the ‘antidisease vaccine’ context of the study.

In summary, cattle in the Quil A™, FCA and GERBU™ groups showed the highest antibody response. A short lived ‘booster effect of infection’ was observed in the GERBU™ and Quil A™ groups while the highest level of *in-vitro* inhibition of C2 activity was observed for the IgGs from the Quil A™ group.

## Discussion

The purpose of this study was to find the best adjuvant(s) giving an adequate antibody response to be used in anti-disease vaccine trials against African trypanosomosis. The ‘best’ adjuvant was chosen based on the magnitude and sustainability of the antibody response, presence of C2 inhibiting antibodies and a ‘booster effect of infection’ but the cost factor, in case of later up-scaling could not be neglected. Purified recombinant C2 was used as the model for this study and its dimeric conformation was assessed thoroughly before use [16]. Recent studies show that C2 protective epitopes seem to be dimer associated [16], hence it is important that the adjuvant preserve the conformation of the epitopes *i.e.* the adjuvant should ideally have a neutral pH and not denature the immunogen.

Quil A™, a saponin-based adjuvant, was the best adjuvant when the above mentioned parameters were considered as well as ease of mixing with the antigen prior to immunization. Quil A™ is purified from a bark extract of *Quillaja saponaria* Molina, a tree native to the Andes region [13]. It consists of a triterpenoid aglycone to which a.o. one or more sugar chains are attached [28]. Saponin-based adjuvants have the ability to enhance antibody production as well as stimulate the cell mediated immune system. They also have the advantage that only a low dose is needed for adjuvant activity [29]. Saponins reportedly induce production of cytokines such as interleukins and interferons that might mediate their immunostimulant effects [13]. It is currently unknown if the adjuvant effect of saponins is related to pore formation, which may allow antigens to gain access to the endogenous pathway of antigens presentation, promoting cytotoxic T-lymphocyte response [30]. Other studies also suggest that *Quillaja* saponin has mitogenic activity and induces T and B- cell proliferation [31].

Animals that received C2 with FCA produced an antibody response in both mice and cattle. Composed of mineral oil, surfactant and mycobacteria, FCA has always been considered to be one of the most effective adjuvants. However, in the cattle experiment, the response waned after some time, such that ODs obtained in ELISA measuring antibody levels post infection were not significantly different from those obtained for the C2 alone group (p >; 0.05). Low PCV was observed for the cattle FCA group, indicating that antibodies produced with FCA were clearly not protective. Since C2 contains highly conformational epitopes it is not unlikely that exposure to the lipophilic environment of the oil emulsion induced conformational changes of C2 and subsequent reduction of immunogenicity with time and this could explain why there was no protection and no booster effect post-infection.

GERBU™ owes its adjuvant effect to the solid ultrafiltrable particles of slowly biodegradable lipids (responsible for depot effect) and the synergistic action of N-acetyl-glucosaminyl-N-acetylmuramyl-L-alanyl-D-isoglutamine, a glycopeptide in the cell wall of *Lactobacillus bulgaricus*. Both mice and cattle immunized with C2 in GERBU™ showed a good antibody response. In cattle, the response was significantly higher than for the FCA group post challenge (p <; 0.05).

Comparative studies in humans and animals have shown that aluminum is a weak adjuvant for antibody induction against small sized recombinant protein vaccines [32]. In the present study however, Adjuphos™ and Alhydrogel™- administered groups showed good antibody responses in mice. Furthermore, the mouse group administered with Adjuphos showed a booster effect of infection. Animals in the Adjuphos-administered group produced a significantly higher response (p <; 0.05) than those in the Alhydrogel group possibly because the antigen could, for some reason have had a higher binding to the phosphate than to the hydroxide form.

Partial protection was observed in cattle immunized with C2 in Quil A™ and in GERBU™. The high level of inhibition of *in-vitro* C2 activity by IgGs from cattle immunized with C2 in Quil A™ or GERBU™ is an indicator of the beneficial effect of these adjuvants in stimulating the production of protective antibodies. Antibodies that show anti-C2 activity *in-vitro* may have the ability to inhibit the deleterious effects of circulating congopain *in-vivo* such as immunosuppression, which may aid in reducing the pathological effects of trypanosomosis [12]. There was however no correlation between the C2 inhibition activity and capacity to prevent a decrease in PCV for the adjuvant groups.

Although this study evaluated the anti-C2 response using antibody parameters, the safety of the adjuvants is also an important parameter to consider. In our study, no adverse effects were observed for most of the adjuvants, a notable exception is the mice immunized with C2 in FCA, of which 7 out of 10 died before the time of challenge with trypanosomes. In cattle, one of the animals in the group that was immunized with FCA showed an inflammatory response with formation of an open necrotic wound. Two other animals in this group showed painful swellings lasting several days. Furthermore, immunization parameters can also affect response *i.e.* adjuvant and antigen dose, route and schedule of immunization.

## Conclusions

We have established that the use of purified saponin Quil A™ enables the induction of a strong, sustained antibody response against congopain (C2) in cattle. This antibody response is also partially protective, purportedly due to the anti- congopain antibodies produced by the immunized animals, which inhibit congopain activity. This investigation shows that use of a recombinant antigen representative of the type of molecules that is to be utilized in the design of a multicomponent anti-disease vaccine (typically active enzymes), enabled the identification of the most suitable adjuvant for this type of study. This will make it possible to conduct vaccine trials devoid of the artifacts associated with an inadequate immune response, to focus solely on the effects of the studied antigens. It is hoped that it will serve as a stepping stone to allow the development of an effective anti-disease vaccine against African trypanosomosis.

## Abbreviations

AMC: 7-amino 4-methyl coumarin; DTT: Dithiothreitol; E-64: L-trans-epoxysuccinyl-leucylamido(4-guanidino)butane; FCA: Freund’s complete adjuvant; FIA: Freund’s incomplete adjuvant; HRPO: Horse radish peroxidise; OPD: Ortho-phenylenediamine; OD: Optical density; PBS: Phosphate buffered saline; PCV: Packed cell volume; Z: Benzyloxycarbonyl.

## Authors' contributions

JK conducted the experiments described in the manuscript; GWL, EBL and THTC are the co-investigators, assisting in experimental design and interpretation of results and editing of the manuscript. EA initiated work on the role of adjuvants in immunogenicity of congopain. AFVB is the principal investigator who oversaw the study and manuscript writing. All authors read and approved the final manuscript.
